# Expression of Alzheimer-Type Neurofibrillary Epitopes in Primary Rat Cortical Neurons Following Infection with *Enterococcus faecalis*

**DOI:** 10.3389/fnagi.2015.00259

**Published:** 2016-01-20

**Authors:** Robert Underly, Mee-Sook Song, Gary L. Dunbar, Charles L. Weaver

**Affiliations:** ^1^Department of Psychology, Saginaw Valley State UniversityUniversity Center, MI, USA; ^2^Field Neurosciences InstituteSaginaw, MI, USA; ^3^Department of Psychology, Central Michigan UniversityMount Pleasant, MI, USA; ^4^Department of Health Sciences, Saginaw Valley State UniversityUniversity Center, MI, USA

**Keywords:** Alzheimer’s disease, *Enterococcus faecalis*, edentulism, chronic periodontitis, tau, phosphorylation, Alz-50

## Abstract

The neurofibrillary tau pathology and amyloid deposits seen in Alzheimer’s disease (AD) also have been seen in bacteria-infected brains. However, few studies have examined the role of these bacteria in the generation of tau pathology. One suggested link between infection and AD is edentulism, the complete loss of teeth. Edentulism can result from chronic periodontal disease due to infection by *Enterococcus faecalis*. The current study assessed the ability to generate early Alzheimer-like neurofibrillary epitopes in primary rat cortical neurons through bacterial infection by *E. faecalis*. Seven-day old cultured neurons were infected with *E. faecalis* for 24 and 48 h. An upward molecular weight shift in tau by Western blotting (WB) and increased appearance of tau reactivity in cell bodies and degenerating neurites was found in the 48 h infection group for the antibody CP13 (phospho-Serine 202). A substantial increase in reactivity of Alz-50 was seen at 24 and 48 h after infection. Furthermore, extensive microtubule-associated protein 2 (MAP2) reactivity also was seen at 24 and 48 h post-infection. Our preliminary findings suggest a potential link between *E. faecalis* infection and intracellular changes that may help facilitate early AD-like neurofibrillary pathology.

**Highlights**
- *Enterococcus faecalis* used in the generation of AD neurofibrillary epitopes in rat.
- Infection increases Alz-50, phospho-Serine 202 tau, and MAP2 expression.
- Infection by *Enterococcus* may play a role in early Alzheimer neurofibrillary changes.

## Introduction

Neurofibrillary tau pathology and beta amyloid deposition are the classic defining biological markers of Alzheimer’s disease (AD). The appearance and spread of neurofibrillary tangles and amyloid plaques have been shown to correlate with the onset and severity of Alzheimer-type cognitive decline (Braak and Braak, 1995; Sperling et al., 2011). Although these pathological hallmarks have been studied extensively, a mechanism for their onset in AD has yet to be elucidated. During the past three decades, studies have re-examined the presence of bacterial infection in AD brains. Various types of spirochetes, including six periodontal pathogen spirochetes and *Borrelia burgdorferi* (MacDonald, 1986; MacDonald and Miranda, 1987; Miklossy, 1993; Riviere et al., 2002; Miklossy et al., 2004) and an obligate intracellular bacterium *Chlamydophila (Chlamydia) pneumoniae* (Balin et al., 1998, 2008; Hammond et al., 2010) have been detected and isolated from Alzheimer brain tissue, yet few studies have examined the involvement of these pathogens in the production of AD-like tau/neurofibrillary pathology (Miklossy et al., 2006).

The relationship between chronic periodontitis and AD has been shown with respect to periodontal pathogens (*Treponema denticola, Tannerella forsythia*, and *Porphyromonas gingivalis*) and their virulence factors (Poole et al., 2013, 2015; Singhrao et al., 2014). Infection from *Enterococcus faecalis*, the bacteria most commonly associated with failed endodontic procedures (Sundqvist et al., 1998; Rocas et al., 2004; Zoletti et al., 2006; Al-Ahmad et al., 2010), secondary endodontic infections (Schirrmeister et al., 2009), urinary tract infections (Zoletti et al., 2006) and nosocomial infection (Courvalin, 2006), also has been implicated in the pathogenesis of chronic periodontitis (Sun et al., 2012). These bacteria can form biofilms, making them extremely difficult to remove and allowing for cell microcolony formation which results in chronic infection (Mohamed and Huang, 2007; Arciola et al., 2008). Edentulism, the complete loss of teeth, also has been shown to be present in a subset of individuals with AD or cerebral atherosclerotic pathologies (Gallo et al., 2005); and has chronic periodontitis as its most common factor. Lastly, it has been suggested that *E. faecalis* can travel from sites of dental infection to the brain (Mylona et al., 2012); thus making it an attractive candidate associated with AD pathology.

To address if early AD-like neurofibrillary epitopes could be generated by infection with *E. faecalis*, we examined potential posttranslational modifications of tau in primary rat cortical neuron cell cultures after infection with *E. faecalis*. Antibodies recognizing conformational change (Alz-50) and phosphorylation (CP13) of tau were used to detect these epitopes after the addition of bacteria. This study demonstrates that *E. faecalis* increases both Alz-50 and CP13 reactivity, as well as that of microtubule-associated protein 2 (MAP2), in cortical cultured neurons; and provides support for the hypothesis that bacterial infection may play a potential role in Alzheimer pathology.

## Materials and Methods

### Bacterial Preparation and Primary Neuronal Cultures

*Enterococcus faecalis* (ATCC 29212) was cultured in 7 ml of brain-heart infusion (BHI) broth in 15 ml conical tubes, and was maintained in an incubator, separate from the neuronal cultures. The bacteria were stored at 37°C in a 5% CO_2_-humidified incubator. Primary cortical neurons from day-18 Fisher 344 rat embryos (Cat. #A10840, Gibco/Invitrogen) were cultured on poly-d-lysine coated 6- and 12-well cell culture plates to a density of 1.7 × 10^5^ and 8.3 × 10^4^ per well, respectively. Neurons were plated using Neurobasal medium with 2% B27, 1% Penicillin-Streptomycin, 0.5% fetal goat serum (FGS), and 0.5 mM GlutaMax (Cat. #35050, Gibco/Invitrogen). Cells were grown at 37°C in a 5% CO_2_-humidified incubator with media being changed every 3–4 days. Seven-day old cultured neurons were infected with *E. faecalis*, with the final concentration of bacteria per well being 7 × 10^6^ bacteria/ml. Neurons were harvested for immunohistochemistry (IHC) and Western blotting (WB) at 24 and 48 h post-infection time points. Experiments were run in triplicate.

### Antibodies

Primary antibodies recognizing pathological changes in tau (Alz-50, conformation; and CP13, phospho-Serine 202; generous gift from Dr. Peter Davies, Albert Einstein College of Medicine) were used for IHC and WB. Anti-β-tubulin (Cat. # ab5392, Abcam) and anti-MAP2 (Cat. #ab56676, Abcam) primary antibodies were used as controls in WB and IHC, respectively. The anti-tubulin antibody was used to standardize protein levels; and for lack of availability of a suitable commercial rat-specific pan-tau antibody that provides consistent staining of tau. Horseradish peroxidase (HRP)-labeled secondary antibodies (Southern Biotech, Birmingham, AL, USA) were used for WB, and fluorescence-labeled secondary antibodies (Molecular Probes, Invitrogen) were used for IHC.

### Western Blot

Cell lysates were analyzed by SDS–PAGE and Western blot. Briefly, cells were washed with tris-buffered saline (TBS) and solubilized in ice-cold lysis buffer (50 mM Tris, pH 7.4, 5 mM EDTA, 150 mM NaCl, 1 × ProteaseArrest and 1 × PhosphataseArrest [GBiosciences, St. Louis, MO, USA]) containing 1% SDS. Cells immediately were scraped off the plates and transferred to a microcentrifuge tube. After centrifugation, sample buffer was added to the supernatant in an equal volume, and the entire sample was heated at 100°C for 5 min. Proteins were separated in SDS-PAGE (10% polyacrylamide gels; Bio Rad, Hercules, CA, USA) and transferred onto 0.45 μm nitrocellulose membranes. Membranes were blocked in 5% fat-free milk in TBS (milk), and incubated with primary antibodies overnight at 4°C (1:200 for Alz-50, CP13, and tubulin). An enhanced and optimized 4-chloronaphthal (Opti-4CN, Cat. # 170-8235, Bio Rad) detection procedure was then used. Procedures for the non-infection group were always performed first to avoid accidental contamination.

### Immunohistochemistry

A general IHC double-labeling technique was performed using the previously mentioned primary antibodies. Cells plated in 12-well culture plates (one control plate and one infected plate) with poly-d-lysine coated coverslips were fixed using 10% buffered formalin. Anti-tau primary antibodies (Alz-50 and CP13; 1:200 dilution) and anti-MAP2 (control; 1:1000 dilution) were added individually to their specified wells and allowed to incubate at 4°C for 24 h. After washes in TBS, primary antibodies were removed and secondary antibodies were added in a dark room to avoid loss of fluorescence. Secondary antibodies (1:500 dilution for all) were removed, and after additional washes in TBS, coverslips were mounted and prepared for confocal fluorescent microscopy analysis (Olympus FluoView FV10i).

### Total Volume Quantification of Alz-50

Imaging of antibody-labeled cells was performed using an Olympus confocal microscope and FV-1000 software. Due to the presence of Alz-50-positive cells in young rat brain (Byne et al., 1991), a method for determining total volume of Alz-50 expression, with or without infection, was needed. Volume images from the confocal 3D data sets were processed with IMARIS software (Bitplane AG, Zurich, Switzerland). Briefly, in order to avoid bias in the volume quantification, regions of interest were selected on a similar grid pattern with the same number and location of regions for each slide being analyzed. Images were opened individually and within the z-stack image, a position 1/3 of the way through the stack was selected. This position (field) remained constant for all images being analyzed. After the appropriate fluorescence channel was selected, absolute and lower threshold intensities were set. Volume data were collected through the entire cell by the software, and values given in μm^3^. Data were exported into Microsoft Excel, and used in an independent samples *t*-test. Files were coded so that groups were not known to the researcher performing the analysis.

## Results

### Increased Alz-50, CP13, and MAP2 Reactivity Following Infection

**Figure 1:**
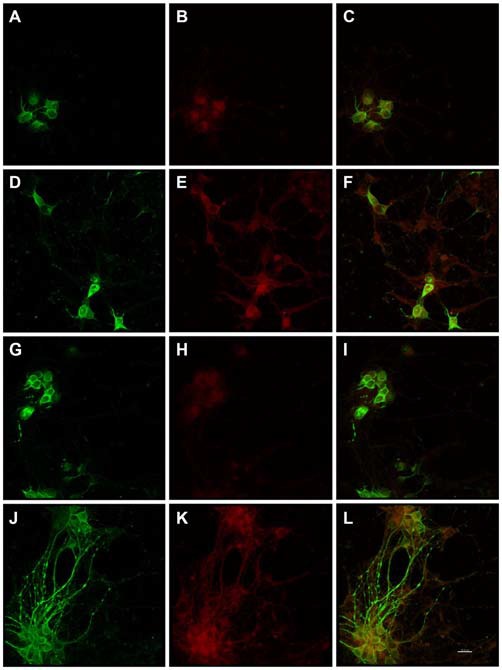
**Substantially increased amounts of Alz-50 and MAP2 reactivity were induced in primary rat cortical neurons by exposure to *Enterococcus faecalis*.** MAP2 (control-green) and Alz-50 (red) staining can be seen in both 24 h **(A–C)** and 48 h **(G–I)** control cultures (**C,I**, co-localization). Alz-50 reactivity was apparent in the cell bodies of neurons in control cultures, and confirms that its expression can be found in rat neurons. A striking increase in Alz-50 reactivity was found at both 24 and 48 h post-infection (**E,K** respectively). Extensive MAP2 reactivity also was seen 48 h post-infection **(J)**, but not at 24 h post-infection **(D)**. Co-localization of MAP2 and Alz-50 staining at 24 and 48 h post-infection is shown in **(F,L)**, respectively. The beaded neurites shown in **(J–L)** are abundant, and may be indicative of degeneration. Scale bar in **L** = 20 μm.

**Figure 2:**
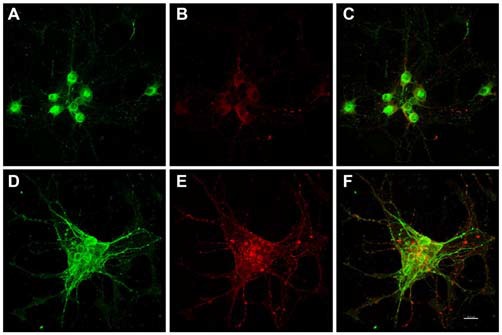
***In vitro* phospho-Serine 202 expression (CP13 reactivity) was increased in primary rat cortical neurons 48 h after exposure to *E. faecalis*.** MAP2 and CP13 reactivities were seen in the cell body of neurons in control cultures (**A,B**, respectively), with co-localization demonstrated in **(C)**. Robust CP13 staining was found throughout the cell bodies and degenerating neurites of neurons post-infection **(E)**. Increased immunoreactivity of MAP2 in infected neurons compared to control cells was also revealed **(D)**. MAP2 and CP13 post-infection co-localization is shown in **(F)**. CP13 reactivity was lost 24 h post-infection (data not shown). Scale bar in **F** = 20 μm.

**Figure 3:**
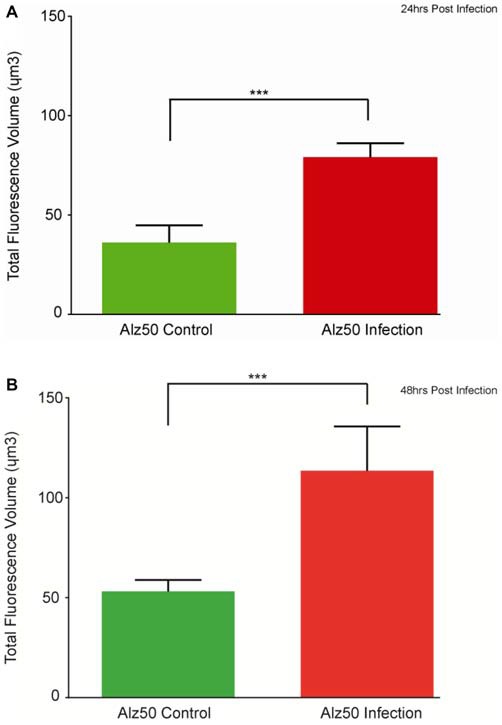
**The appearance of Alz-50 labeling of neurons in control cultures necessitated a comparison of intensities found between control and infected plates.** Alz-50 staining is found in both control and infected cultures, with significantly more reactivity found at both 24 **(A)** and 48 h **(B)** after the introduction of the *E. faecalis*. ^***^Represents *p* < 0.001 **(A)**, *p* = 0.003 **(B)**.

Immunocytochemistry revealed Alz-50 and CP13 reactivity in cell bodies of neurons in control cultures. Alz-50 and CP13 reactivity increased after the introduction of *E. faecalis* in primary rat cortical neuron cultures. Numerous degenerating (beaded) neurites as well as whole cell bodies were labeled with Alz-50, both 24 and 48 h post-infection (Figure 1). Immunostaining revealed similar changes in the location and abundance of CP13 labeling 48 h post-infection (Figure 2). There was nearly complete absence of CP13 labeling at 24 h. MAP2 immunoreactivity of many neurons was also increased at 48 h post-infection compared to control cells. An independent samples *t-test* was used to determine the difference in amounts of Alz-50 reactivity between control and infected cells at two time points (Figure 3). Analyses demonstrated a significant increase (*p* < 0.001; Fields examined: control *N* = 14, infection *N* = 21) in Alz-50 reactivity 24 h post-infection when compared to the 24 h control. A significant increase (*p* = 0.003; Fields examined: control *N* = 24, infection *N* = 15) in Alz-50 reactivity was also demonstrated 48 h post-infection, when compared to the 48 h control.

### Mobility Shift in Phospho-Tau after Infection

**Figure 4:**
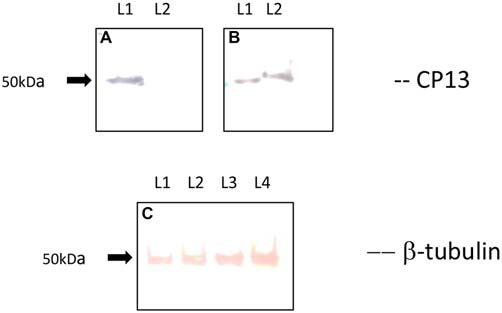
**Immunoblot of CP13 and tubulin reactivity in primary rat cortical neurons.** CP13 staining of control samples reveals a tau isoform around 50 kDa (**A,B**; lane 1 [L1]). There is a distinct loss of CP13 reactivity in samples at 24 h post-infection (**A**; lane 2 [L2]). Blotting of samples at 48 h post-infection demonstrates an upwards shift in mobility of CP13-reactive tau (**B**; lane 2 [L2]) . The shift in mobility may be the result of increased phosphorylation leading to conformational changes in the protein which causes delayed migration through the gel. β-tubulin was used as a loading control for the amount of protein loaded per lane **(C)**. L1 and L2 reveal β-tubulin staining from control and 24 h post-infection samples, while L3 and L4 reveal β-tubulin staining from control and 48 h post-infection samples.

Western blot analysis revealed a loss of CP13 reactivity at 24 h post-infection, and a restoration of reactivity with an upwards molecular weight shift in tau at 48 h post-infection (Figure 4). A similar shift was not seen with the Tubulin control. Immunoblotting failed to yield any reactivity using Alz-50.

## Discussion

Spirochetes, a phylogenetically distinct group of bacteria, have been detected and isolated in Alzheimer brain tissue. Several types of spirochetes were detected in the AD brain (reviewed by Miklossy, 2011a), including six periodontal pathogen spirochetes: *Treponema (T). denticola, T. socranskii, T. pectinovorum, T. amylovorum, T. maltophilum, and T. medium* (Riviere et al., 2002) and the Lyme disease spirochete *B. burgdorferi* (MacDonald and Miranda, 1987), which was shown to induce β-amyloid (Aβ) deposits and increased level of phosphorylated tau successfully in neuronal cell cultures (Miklossy et al., 2006). *In vivo* studies using intranasal injection of an obligate intracellular bacterium such as *C. pneumoniae* have been shown to induce beta amyloid deposition in the brains of BALB/c mice (Little et al., 2004). More recently, additional association between periodontal pathogens and AD has been demonstrated (Poole et al., 2013, 2015; Singhrao et al., 2014), along with the association between chronic periodontitis and amyloid load (Kamer et al., 2015). Several extensive reviews of the literature have also reinforced the significant association between AD and detectable evidence of infection (Miklossy, 2011b; Maheshwari and Eslick, 2015; Shoemark and Allen, 2015). Together, these studies suggest a correlation between Alzheimer’s pathology and bacterial infection in brain. Pertinent to the present study, a potential link between oral health and AD has been reviewed by Azaroazhooh et al. (2010). A study by Gatz et al. (2006) demonstrated that substantial tooth loss prior to the age of 35 was a significant risk factor for the development of dementia and AD. Two studies suggested that edentulous patients, not using dentures, were at higher risk for either dementia (Kim et al., 2007) or mortality (Shimazaki et al., 2001). An inverse association was also found between having a low number of teeth and dementia with an absence of the ApoE4 allele (Stein et al., 2007). These studies, along with the personal observation of numerous edentulous AD patients, ultimately led to the examination of whether the powerful dental pathogen, *E. faecalis*, might induce the biological markers of AD *in vitro*, which would indicate the potential role of this bacterium in AD pathology. The authors concede, that in the case of positive results, future studies showing more direct evidence of the link between the two is needed. Examination for the remnants of *E. faecalis* in brains of edentulous and non-edentulous (with severe on-going periodontal disease) AD patients obviously is the first step; and human AD tissue recently has been secured for analysis by the authors. Of particular interest would be those who experienced edentulism due to advanced caries, failed root canal therapy, or untreatable/refractory periodontal disease since enterococci have been demonstrated in these ailments (Kinsel and Lamb, 2001; Sedgley et al., 2004, 2005; Balaei-Gajan et al., 2010; Kouidhi et al., 2011). Nevertheless, the present study further suggests that agents involved in the pathogenesis of chronic periodontal disorders and edentulism may be related to Alzheimer’s pathology; since *E. faecalis in vitro* can influence the production of pathological epitopes similar to that seen early in AD.

Antibodies recognizing conformational change (Alz-50) and specific phosphorylations of tau protein (CP13; phospho-Serine 202) have been shown to label pre-tangle neurons; and therefore, are suitable markers of early signs of Alzheimer’s degeneration (Jicha et al., 1997; Weaver et al., 2001; Andorfer et al., 2003, 2005). IHC, performed post-infection, revealed morphological changes in the form of neuritic beading, as well as the increased presence of Alz-50 and CP13 reactivity throughout the entire cell. Because of *E. faecalis’* strong virulence, infected neurons tend not to survive in culture much longer than 60 h; so these neurons were harvested at 24 and 48 h post-infection. No changes in reactivity were seen in control samples, which were maintained in culture for 4 weeks.

### The Nature of Alz-50 Reactivity

Alz-50 has been shown to label limited cell populations in healthy young rat tissue (Byne et al., 1991) which led to the expectation of finding some Alz-50 reactivity in control primary rat cortical neurons. Using quantitative total volume analysis, the amount of reactivity was deciphered in both the infection and control groups. These data reveal a significant post-infection increase in Alz-50 reactivity at both 24 and 48 h time points. This study provides no evidence that bacterial infection leads to greater amounts of conformational changes of tau. It does, however, confirm the utility of Alz-50 as a marker of epitopes normally found in early AD-like degeneration. Al-Ghoul and Miller (1989) demonstrated Alz-50 labeling in rat neonatal subplate and cortical neurons prior to their elimination from the cortex. A subsequent paper from the same group showed Alz-50 expression in the principal sensory nucleus following trigeminal nerve lesion (Miller et al., 1994). These studies suggest that Alz-50 reactivity may be a marker for neuronal death. A possible substrate reacting with Alz-50 is the fetal Alz-50 clone 1 (FAC1) protein. Previous studies have demonstrated that FAC1 is in abundance in the cell bodies and dendrites of human undifferentiated neurons (Bowser et al., 1995), and have shown an upregulation in FAC1 expression after lesioning of intact rodent tissue (Styren et al., 1997). In the present study, we did not observe Alz-50 reactivity by immunoblotting; and in fact, achieving Alz-50 reactivity when using unpurified and unconcentrated primary cortical rat cell lysate has proven difficult (unpublished raw data). The authors are only familiar with one study demonstrating significant Alz-50 reactivity from rat neurons by immunoblotting; and it was achieved after isolation of the antigen by immunoaffinity chromatography and subsequent concentration (Al-Ghoul and Miller, 1989). Regardless of whether the protein involved is tau or FAC1, it is clear that increased Alz-50 reactivity with the protein is associated with degeneration of the neurons.

### Phosphorylation Changes after Infection

The CP13 reactivity seen by immunoblotting in control cell cultures suggests that Serine 202 is normally phosphorylated in a portion of primary rat cortical neurons. The loss of reactivity at 24 h and the recovery of signal at 48 h post-infection together were surprising. However, studies have shown that oxidative stress, in response to ischemia, can induce rapid dephosphorylation of tau (Shackelford and Yeh, 1998), with recovery of the epitope equally rapid for Serine 202/Threonine 205 (Mailliot et al., 2000). Recent studies, also, have demonstrated the ability of *E. faecalis* to produce oxidative stress through the generation of reactive oxygen species (Golińska et al., 2013; Strickertsson et al., 2013, 2014). Therefore, it may be possible that the bacteria caused local ischemic conditions in the culture dish. Since CP13 is a phosphorylation- and sequence-dependent antibody, dephosphorylation at Serine 202 will result in loss of reactivity. [Note: C.Weaver is the co-generator of the CP13 antibody with P. Davies] The upward molecular weight shift of the tau band in the infection cultures suggests an increase in phosphorylation of tau that may lead to conformational changes that effectively reduces its migration through the acrylamide gel (Lindwall and Cole, 1984; Bretteville et al., 2009). Phosphorylation at one site (Ser 202) may not be enough to generate conformational change in tau. However, it is likely that multiple sites are being phosphorylated since glycogen synthase kinase 3 Alpha and Beta (GSK3α and 3β), microtubule-associated protein kinase 13 (MAPK13), cyclin-dependent kinase 5/p25 (CDK-5/p25), and a host of others can simultaneously phosphorylate Ser 202, Thr 231, Ser 235, and Ser 396/404 (Cavallini et al., 2013). The authors only demonstrated one tau band at 48 h post-infection. It is possible that there would be two bands of tau after re-phosphorylation and subsequent increased phosphorylation. The primary author’s research facilities did not house a film developer, so enhanced chemilluminescence (ECL) was not utilized. The Opti-4CN used in the present study may not have been sensitive enough to display a second tau band similar to that found from control samples. Along with the increase in immunostaining throughout the neurites, it is clear that CP13 reactivity increased as a result of infection. To optimize material for immunoblotting, volume analyses were not performed for CP13 reactivity.

### MAP2 and Neurodegeneration

The use of MAP2 as a control marker for primary rat neurons is well documented. Increases in MAP2 staining post-infection were not expected, but are not unreasonable. In AD tissue, MAP2 staining can be found in dystrophic neurites (Neve et al., 1986; Trojanowski et al., 1991; Ashford et al., 1998). While protein levels of MAP2 do not change significantly in AD, its immunoreactivity in rat neuronal cell cultures has been shown to increase in response to serum from AD patients, particularly at 48 h post-application (Brewer and Ashford, 1992). The results of increased MAP2 reactivity and morphological changes to neurites, in this study, provide support for neuritic reorganization and degeneration in this cell culture model.

### Aβ_42_ and *Enterococcus faecalis*

A recent study has shown that Aβ_42_ functions as an antimicrobial peptide (AMP) that expresses activity against *E. faecalis* (Soscia et al., 2010). The group demonstrated that Aβ_42_ was present at significantly greater levels in the temporal lobe of AD patients compared to age-matched non-AD brains. These findings suggest that increased levels of Aβ_42_ in AD brain tissue could be due to an immune response of Aβ_42_ to an infection-inducing microorganism present in the tissue. These data lend support to the idea that the pathology seen in AD could be related to an infectious agent.

## Conclusion

These preliminary data suggest that pathologic epitopes generated in early AD may be influenced by infection with *E. faecalis*. While the nature of the type of protein eliciting Alz-50 reactivity is unknown, it is important to recognize that reactivity is significantly increased after infection. As a result, further research on the implications of bacterial infection and AD is warranted.

## Author Contributions

RU, performed all aspects of the experiments, and wrote the manuscript. CLW, organized, designed, and performed all parts of the experiments; and edited the manuscript. M-SS, performed the cell culture work. GLD, helped to oversee the cell culture work; and edited the manuscript.

## Conflict of Interest Statement

The authors declare that the research was conducted in the absence of any commercial or financial relationships that could be construed as a potential conflict of interest.
